# Gratuitous Hint for Advertising Doctors

**Published:** 1878-02

**Authors:** 


GRATUITOUS HINT FOR ADVERTISING DOCTORS.—Some ingenious person has hit upon a plan for providing food for the mind and body simultaneously. This is to be achieved by having advertisements printed upon refreshment-bar crockery. For this "The Continental Advertising Refreshment Plate Company" ask for £5,000 from the public, the total capital proposed being £10,000. The method projected is, we are told, "by neatly designed advertisements on the rims of refreshment plates, dishes, saucers, etc., which are to be made of porcelain, china, earthenware, or other material, and to be distributed among the different hotels, restaurants, and cafés in the chief cities and towns of France and Belgium." This is to be "the cheapest mode of permanent advertising ever yet introduced," as so many plates will be seen by so many people per day, and "the average cost to advertisers will be about 1¾d. per plate for each advertisement." What next?—*American Bi-Weekly.*

